# Mechanisms of group A *Streptococcus* resistance to reactive oxygen species

**DOI:** 10.1093/femsre/fuu009

**Published:** 2015-02-10

**Authors:** Anna Henningham, Simon Döhrmann, Victor Nizet, Jason N. Cole

**Affiliations:** 1 Department of Pediatrics, University of California San Diego, La Jolla, CA 92093, USA; 2 The School of Chemistry and Molecular Biosciences, The University of Queensland, St Lucia, QLD 4072, Australia; 3 The Australian Infectious Diseases Research Centre, The University of Queensland, St Lucia, QLD 4072, Australia; 4 Skaggs School of Pharmacy and Pharmaceutical Sciences, University of California San Diego, La Jolla, CA 92093, USA; 5 Rady Children's Hospital, San Diego, CA 92123, USA

**Keywords:** Group A *Streptococcus*, innate immunity, oxidative stress resistance, reactive oxygen species, *Streptococcus pyogenes*, virulence

## Abstract

*Streptococcus pyogenes*, also known as group A *Streptococcus* (GAS), is an exclusively human Gram-positive bacterial pathogen ranked among the ‘top 10’ causes of infection-related deaths worldwide. GAS commonly causes benign and self-limiting epithelial infections (pharyngitis and impetigo), and less frequent severe invasive diseases (bacteremia, toxic shock syndrome and necrotizing fasciitis). Annually, GAS causes 700 million infections, including 1.8 million invasive infections with a mortality rate of 25%. In order to establish an infection, GAS must counteract the oxidative stress conditions generated by the release of reactive oxygen species (ROS) at the infection site by host immune cells such as neutrophils and monocytes. ROS are the highly reactive and toxic byproducts of oxygen metabolism, including hydrogen peroxide (H_2_O_2_), superoxide anion (O_2_•^−^), hydroxyl radicals (OH•) and singlet oxygen (O_2_*), which can damage bacterial nucleic acids, proteins and cell membranes. This review summarizes the enzymatic and regulatory mechanisms utilized by GAS to thwart ROS and survive under conditions of oxidative stress.

## Abstract (precis)

This review discusses the mechanisms utilized by the bacterial pathogen group A *Streptococcus* to detoxify reactive oxygen species and survive in the human host under conditions of oxidative stress.

## Graphical abstract

**Graphical Abstract Figure.**
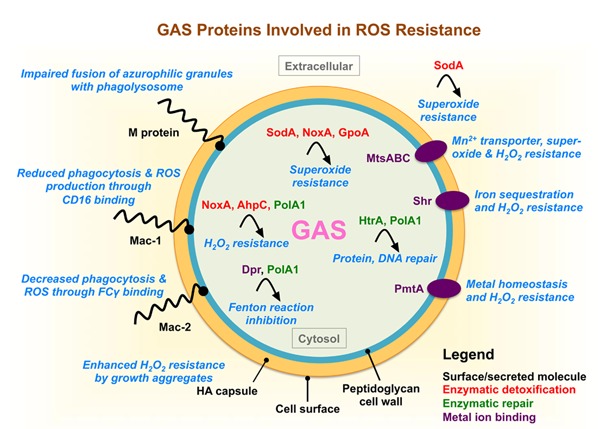
This review discusses the mechanisms utilized by the bacterial pathogen group A *Streptococcus* to detoxify reactive oxygen species and survive in the human host under conditions of oxidative stress.

## INTRODUCTION

Reactive oxygen species (ROS) are highly reactive oxygen-containing molecules such as oxygen ions and peroxide generated from the metabolism of oxygen. Exogenous ROS can be formed following exposure to physical or chemical agents such as ionizing radiation (Dainton 1948), ultraviolet (UV) radiation (Jurkiewicz and Buettner 1994), mitomycin C (Tomasz 1976) or desiccation (Potts 1994). Depending on the setting, and the concentration, ROS can be either essential for, or detrimental to, cellular survival. ROS is required for mammalian cell survival, growth, proliferation and differentiation (Droge 2002), notably through its role in intracellular signaling (Finkel 1998; Rhee 1999); however, ROS can also be highly injurious to cells, causing damage to proteins and DNA (Brawn and Fridovich 1981), provoking lipid peroxidation (Niki 2009) or oxidizing enzyme cofactors. ROS play a role in the development of a number of human diseases, including chronic inflammation, age-related disorders and cancers (Liou and Storz 2010). Host phagocytic cells, primarily neutrophils and monocytes generate endogenous ROS to facilitate clearance of bacteria at the infection site.

Bacterial cells synthesize a number of enzymes to detoxify ROS, including alpha-1-microglobulin (Olsson *et al.*, 2012), superoxide dismutases (Sods) (Lynch and Kuramitsu 2000), catalases (Mishra and Imlay 2012), glutathione peroxidases (Moore and Sparling 1995) and peroxiredoxins (generally referred to as AhpC in bacteria) (Poole 2005; Dubbs and Mongkolsuk 2007). Antioxidants, including ascorbic acid (vitamin C), tocopherol (vitamin E), uric acid and glutathione, enhance resistance to oxidative stress. Reactive nitrogen species (RNS) are another group of antimicrobials produced by certain immune cells, resulting in nitrosative stress. RNS are most often derived from nitric oxide and superoxide. Like ROS, RNS have diverse functions within the host, and the importance of RNS in health and disease has been reviewed elsewhere (Nathan and Shiloh 2000; Fang 2004) and is not a focus of this review.

Bacterial pathogens such as *Streptococcus pyogenes*, also known as group A *Streptococcus* (GAS), encounter ROS during the innate immune response driven by host phagocytic cells. GAS is a Gram-positive facultative anaerobe, currently listed as a ‘top 10’ human pathogen, responsible for 700 million infections annually (Carapetis *et al.*, 2005). Of these infections, 1.8 million are severe invasive infections (bacteremia, toxic shock syndrome and necrotizing fasciitis) with a mortality rate of 25%. The remaining infections caused by GAS are benign and self-limiting and include pharyngitis and impetigo (Walker *et al.*, 2014). Currently, there is no safe and efficacious vaccine to protect against GAS infection (Henningham, Gillen and Walker 2013). A better understanding of GAS pathogenesis and the complex interplay between GAS and host proteins, molecules and tissues, may lead to the development of novel therapeutic agents or effective vaccines. One group of host defense factors of particular interest in this regard is ROS. GAS encounters ROS at various stages of infection, and consequently GAS are well equipped to deal with oxidative stress and express an array of proteins and virulence factors to survive under these harsh conditions. In contrast to the human pathogen *Staphylococcus aureus*, which produces a similar spectrum of skin and invasive infections, GAS lack catalase, a heme-containing peroxidase that degrades hydrogen peroxide (H_2_O_2_) to H_2_O and oxygen (O_2_), and do not express an antioxidant carotenoid pigment to withstand ROS-mediated killing (Liu *et al.*, 2005). Despite this, GAS can tolerate the high oxygen concentrations within the human host and has evolved multiple mechanisms to resist the toxic effects of ROS produced from the reduction of atmospheric oxygen or the activation of host phagocytic cells (Table 1). Two fundamental mechanisms are employed by GAS to counter ROS: 1) suppression of ROS (indirect), and 2) expression of enzymes to detoxify ROS (direct). This review focuses on the ROS resistance mechanisms of GAS including the expression of surface-associated ROS resistance factors, intracellular and secreted enzymes involved in ROS detoxification or repair of ROS-damaged proteins, metal transporters important for metal ion homeostasis and oxidative stress resistance, and the transcription factors involved in the coordinated regulation of gene expression for GAS survival under conditions of oxidative stress.

**Table 1.** Resistance mechanisms employed by GAS to thwart ROS.

Resistance mechanism	GAS factor	Function
Surface/secreted protein	M protein	Impaired fusion of azurophilic granules with phagolysosome
	HA capsule	H_2_O_2_ resistance by aggregation
	Mac-1	Reduced phagocytosis and ROS production
	Mac-2	Reduced phagocytosis and ROS production
Enzymatic detoxification	SodA	Superoxide resistance
	AhpC	H_2_O_2_ resistance
	GpoA	Superoxide resistance
	NoxA	Superoxide resistance and H_2_O_2_ resistance
Enzymatic repair	HtrA	Protein repair upon ROS damage
	PolA1	DNA repair upon ROS damage, H_2_O_2_ resistance and inhibition of Fenton reaction
Metal ion binding	PmtA	Metal transporter and H_2_O_2_ resistance
	Dpr	Iron sequestration and H_2_O_2_ resistance
	MtsABC	Metal transporter, superoxide and H_2_O_2_ resistance, inhibition of Fenton reaction
	Shr	Iron sequestration and H_2_O_2_ resistance

## BIOLOGICAL ROLES OF ROS

ROS are generated during the metabolism of O_2_ and consist of highly toxic and reactive oxygen-containing molecules such as peroxide and oxygen ions. ROS are produced either via the transfer of electrons to O_2_, leading to the production of superoxide (O_2_•^−^), H_2_O_2_ and highly reactive hydroxyl radicals (OH•), or via the transfer of energy to O_2_, which leads to the formation of singlet oxygen (O_2_*) (Yesilkaya *et al.*, 2013). Superoxide damages proteins by oxidizing iron–sulfur clusters within enzymes (Kuo, Mashino and Fridovich 1987; Flint, Tuminello and Emptage 1993). The membrane-permeable H_2_O_2_ can target sulfur atoms in cysteine (Winterbourn and Metodiewa 1999) or methionine residues in proteins (Griffiths and Cooney 2002), and cause oxidative damage to cell membranes or nucleic acids (Gabbianelli *et al.*, 1998). Highly reactive singlet oxygen reacts with proteins, lipids, DNA and RNA (Glaeser *et al.*, 2011). Hydroxyl radicals are formed during the Fenton reaction in which H_2_O_2_ is converted to OH• in the presence of metal ions [H_2_O_2_ + Fe(II) → OH• + OH^−^ + Fe(III)]. The highly reactive OH• targets DNA, attacking deoxyribose residues resulting in nicks in double-stranded DNA which can compromise chromosome fidelity and ultimately lead to cell death (Imlay, Chin and Linn 1988; Rai *et al.*, 2001). Carbonylated proteins are an additional and irreversible byproduct of metal-catalyzed oxidative stress (Amici *et al.*, 1989; Dalle-Donne *et al.*, 2006). Carbonylation often results in loss of protein function and is further reviewed in Dalle-Donne *et al.* (2006). Heavily carbonylated proteins can form aggregates, are resistant to proteasomal degradation and are thought to be associated with certain neurodegenerative disorders (Dalle-Donne *et al.*, 2006). ROS also plays a role in the activation of the inflammasome, an important arm of the innate immune defense, whereby pattern-recognition receptors on host cells identify danger to the host, via recognition of pathogen-associated molecular patterns from microbes and damage-associated molecular patterns released from injured tissue (Bauernfeind and Hornung 2013). The most studied inflammasome is the Nod-like receptor (NLR) family, pyrin domain-containing 3 (NLRP3). The production of ROS is an important upstream event of NLRP3 activation (Tschopp and Schroder 2010). ROS have diverse roles within the host, participating in biological processes including cellular signaling, chemotaxis, antigen cross-presentation, autophagy, mammalian cell growth, proliferation and differentiation, and the adaptive immune response, which have been reviewed elsewhere (Thannickal and Fanburg 2000; Droge 2002; Lam, Huang and Brumell 2010; Yang *et al.*, 2013).

## IMMUNE CELLS THAT GENERATE ROS

**Figure 1.**
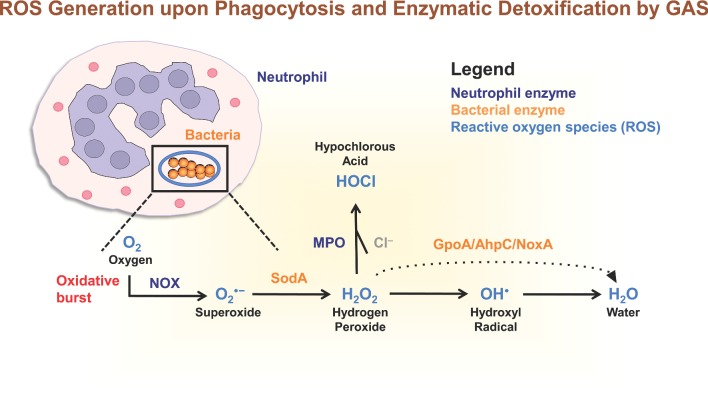
Generation of reactive oxygen intermediates (light blue) by enzymes in human neutrophils (dark blue) upon phagocytosis, and the enzymatic detoxification mechanisms utilized by GAS bacteria (orange). Oxygen (O_2_) can be converted to superoxide (O_2_•^−^) following the activation of NOX in neutrophils. Superoxide may be converted to H_2_O_2_ by the GAS enzyme SodA. Both superoxide and hydrogen peroxide may be converted to hydroxyl radicals (OH•) during the Fenton reaction. Bacteria express iron chelators and transporters to maintain iron homeostasis and prevent the formation of bactericidal hydroxyl radicals. The neutrophil enzyme MPO catalyzes the production of bactericidal HOCl from H_2_O_2_ and Cl^−^ during the oxidative burst. Hydrogen peroxide can be detoxified to water by the enzyme Gpo, AhpC or NoxA. Following the gain of an electron, hydroxyl radicals can be converted to water. Abbreviations: O_2_, oxygen; O_2_•^−^, superoxide; NOX, NADH oxidase; H_2_O_2_, hydrogen peroxide; SodA, superoxide dismutase; OH•, hydroxyl radicals; MPO, myeloperoxidase; HOCl, hypochlorous acid; Cl^−^, chloride anion; GpoA, glutathione peroxidase; AhpC, alkyl hydroperoxidase; NoxA, NADH oxidase A reductase.

It has been long established that during phagocytosis of microbes, phagocytes such as neutrophils and monocytes exhibit increased O_2_ consumption (Sbarra and Karnovsky 1959; Iyer, Islam and Quastel 1961; Rossi and Zatti 1964). This phenomenon is known as respiratory or oxidative burst and is a very important component of innate immunity and the host defense against microbes. A synergy between myeloperoxidase (MPO) (contained within neutrophil azurophilic granules released into the phagosome during the degranulation process), H_2_O_2_ produced by neutrophils, and a halide, often chloride (Fig. 1), results in intracellular killing of Gram-positive and Gram-negative bacterial species (McRipley and Sbarra 1967; Klebanoff 2005). GAS is naturally deficient in catalase and carotenoid pigment expressed by other Gram-positive bacteria such as *Bacillus* spp. (Zuber 2009) and *S. aureus* (Liu *et al.*, 2005), and may be a susceptible target to oxidative burst killing initiated by host cells (Kwinn and Nizet 2007). Specifically, leukocytes release ROS O_2_•^−^ and H_2_O_2_ into the intracellular milieu through the assembly of membrane-bound nicotinamide adenine dinucleotide phosphate (NADH) oxidase 2 (NOX2) on the phagosomal membrane following phagocytosis (Babior, Kipnes and Curnutte 1973; DeLeo *et al.*, 1999; Nauseef 2004). ROS is also generated by the terminal enzyme of purine catabolism, xanthine oxidase (Harrison 2002), the electron transport chain within mitochondria (Murphy 2009; Santos *et al.*, 2009), peroxisomes (Antonenkov *et al.*, 2010) and the endoplasmic reticulum (Santos *et al.*, 2009). There are seven known members of the NOX family, NOX1–5 and DUOX1–2 (Lam, Huang and Brumell 2010), with NOX2 producing the greatest amount of ROS in human tissues. The O_2_•^−^ and H_2_O_2_ produced by NOX2 are subsequently converted to oxidizing radicals (such as OH•) and oxidizing halogens (e.g. hypochlorite, ClO^−^), both of which are powerful microbicidal agents (Babior 1984). The generation of ROS by NOX mediates the formation of neutrophil extracellular traps (NETs) (Fuchs *et al.*, 2007; Bianchi *et al.*, 2009). NETs are released from activated neutrophils and comprise a mixture of granule proteins, antimicrobial peptides and nuclear constituents such as chromatin and histones that combine to form extracellular fibers capable of trapping and killing bacteria (Brinkmann *et al.*, 2004). Therefore, bacteria including GAS have evolved strategies not only to reduce ROS production, but also to further inhibit the generation of NETs and innate immune clearance. GAS resistance to lethal doses of H_2_O_2_ can be induced *in vitro* by growth in the presence of oxygen (Ricci, Janulczyk and Bjorck 2002), 5% ethanol (King, Horenstein and Caparon 2000) or sub-lethal concentrations of H_2_O_2_ (King, Horenstein and Caparon 2000).

## GAS PRODUCES ROS

Certain, but not all, GAS strains are H_2_O_2_ producers when cultured under anaerobic conditions (Saito *et al.*, 2001). Agar-based techniques, involving the Prussian blue forming reaction, have been developed to differentiate H_2_O_2_ producing from non-producing GAS isolates (Saito *et al.*, 2007). The GAS enzymes lactate oxidase (Seki *et al.*, 2004; Kietzman and Caparon 2010) and NOX (Gibson *et al.*, 2000) are responsible for the secretion of up to millimolar concentrations of H_2_O_2_ into the extracellular milieu (Malke *et al.*, 1974; Cleary and Larkin 1979; Gibson *et al.*, 2000; Saito *et al.*, 2001). The reason why GAS generates peroxide is not fully understood. The H_2_O_2_ produced by H_2_O_2_-producing GAS may function to kill other bacterial species in order to help establish infections, as is the case for *Streptococcus pneumoniae* (Pericone *et al.*, 2000) and commensal *Lactobacillus* spp. (Aroutcheva *et al.*, 2001). In invertebrate models of GAS pathogenesis, GAS production of H_2_O_2_ resulted in the death of *Caenorhabditis elegans* (Jansen *et al.*, 2002; Bolm *et al.*, 2004). However, not all GAS isolates causing disease, be it severe or mild, produce H_2_O_2_, and in a study determining H_2_O_2_ production (25 of the 46 clinical strains examined accumulated H_2_O_2_ in the growth medium) there was no correlation between disease state and a H_2_O_2_-producing phenotype (Saito *et al.*, 2001). In this study, non-H_2_O_2_-producing strains were found to be resistant to *in vitro* killing by phagocytes isolated from patients with chronic granulomatous disease (CGD), and *in vivo* subcutaneous injection of H_2_O_2_ non-producing strains resulted in a greater swelling in CGD mice footpads, accompanied by a higher mortality rate, compared to the H_2_O_2_-producing strains (Saito *et al.*, 2001).

## GAS INDUCES ROS

GAS, like other nasopharyngeal pathogens, is exposed to a range of O_2_ concentrations during the infection process, encountering 20% O_2_ on the nasopharynx, 5% O_2_ in the lower respiratory tract and virtually anaerobic conditions in blood (Yesilkaya *et al.*, 2013). During contact with host innate immune cells such as leukocytes and macrophages, GAS induces ROS. In turn, the action of the ROS can result in host cell injury and/or death. For instance, following the production of H_2_O_2_ by host neutrophils, a synergistic interaction between a secreted GAS hemolysin, streptolysin S (SLS) and H_2_O_2_ results in injury to vascular endothelial cells (Ginsburg *et al.*, 1989; Ginsburg and Varani 1993). ROS induced by GAS invasion has also been shown to trigger apoptosis of infected epithelial cells due to mitochondrial dysfunction (Aikawa *et al.*, 2010). Specifically, ROS was produced following activation of the GTPase Rac1 (Aikawa *et al.*, 2010), previously reported to mediate ROS production (Suzukawa *et al.*, 2000).

## SURFACE MOLECULES INVOLVED IN THE OXIDATIVE STRESS DEFENSE OF GAS

One factor contributing to the success of GAS as a human pathogen is its ability to evade and/or defend against the host innate immune response. One such defense mechanism is oxidative stress resistance, for which GAS contain an orchestrated repertoire of surface proteins and polysaccharides to counter the ROS produced by host cells (Table 1).

### M protein

GAS strains are distinguished serologically on the basis of immunovariable surface-anchored M proteins (Facklam *et al.*, 1999). The M protein is a vaccine antigen and major virulence factor of GAS, which affects adherence and invasion to host cells via interaction with multiple host proteins including fibrinogen (Smeesters, McMillan and Sriprakash 2010; Ghosh 2011; Anderson et al., 2014), and inhibits phagocytosis of GAS to overcome innate immunity (Smeesters, McMillan and Sriprakash 2010). There are four known types of granules within neutrophils, the two major granules are the primary (azurophilic) and secondary (specific) granules (Pham 2006). Azurophilic granules contain MPO, and play an active role in the digestion of phagocytosed material, while specific granules are secretory and take part in initiation of the host inflammatory response (Borregaard and Cowland 1997). Live wild-type GAS (expressing M and M-like proteins), after internalization by human neutrophils, increased oxidative burst (specifically H_2_O_2_ production) and membrane traffic responses compared to mutants lacking M protein, specifically via inhibition of the fusion of azurophilic granules with phagosomes (Staali *et al.*, 2006). This is an additional M protein-mediated mechanism of GAS evasion of the host immune system, even once entrapped within phagosomes. Conversely, M1 protein spontaneously released from the surface of GAS or after enzymatic cleavage activates neutrophils as determined by release of heparin-binding protein (Herwald *et al.*, 2004; Macheboeuf *et al.*, 2011). Moreover, M1 protein triggers the release of MPO from neutrophils mediating lung damage (Soehnlein *et al.*, 2008). Additionally, surface-released M1 protein forms a pathological network with fibrinogen, which circulates in high concentrations in human blood (Macheboeuf *et al.*, 2011). This complex contributes to lung damage and inflammation, a condition characterized by high ROS levels (Herwald *et al.*, 2004; Soehnlein *et al.*, 2008; Macheboeuf *et al.*, 2011). Consequently, these findings suggest that the M1 protein released from GAS cell surface may contribute to induction of ROS production; however, further research is required to corroborate the link between M protein and ROS induction (Allen and Stephens 2011).

### Hyaluronan capsule

The surface capsule of GAS is composed solely of hyaluronan (HA); a high-molecular-mass polysaccharide comprised of glucuronic acid and *N*-acetylglucosamine (Kendall, Heidelberger and Dawson 1937). The GAS capsule is structurally identical to the HA widely distributed throughout human tissues, allowing GAS to mimic host structures and thwart detection by the host immune system. The capsule promotes GAS survival by obstructing antibody binding to epitopes on the bacterial surface, complement deposition (Dale *et al.*, 1996) and opsonophagocytosis (Foley and Wood 1959; Dale *et al.*, 1996). The capsule of GAS has also been reported to contribute to resistance against H_2_O_2_. Encapsulated strains grow in aggregates, taking up oxygen at a slower rate than non-encapsulated derivative strains (Cleary and Larkin 1979). HA capsule-mediated aggregation is thought to mechanically shield GAS from destruction by oxygen metabolites such as H_2_O_2_ (Cleary and Larkin 1979).

### GAS Mac-1-like protein (Mac-1/IdeS)

Following a proteomic analysis of GAS culture supernatants, a secreted protein with homology to the α-subunit of human Mac-1, designated the GAS Mac-1-like protein (Mac-1, also known as IdeS), was discovered. Human Mac-1, also known as CDIIb or CD18, is a leukocyte adhesion glycoprotein functioning in cell–cell and cell–substrate adhesive interactions including binding complement product iC3b (Corbi *et al.*, 1988). Consequently, human Mac-1 plays a role in regulating leukocyte migration, phagocytosis and oxidative killing. GAS Mac-1 is thought to function through molecular mimicry by binding CD16 on the surface of neutrophils, consequently inhibiting opsonophagocytosis and production of ROS (Lei *et al.*, 2001). Lei and colleagues further concluded that Mac-1 prevents any receptor–antibody interaction, i.e. activation by IgG binding as an immune suppression strategy by GAS (Lei *et al.*, 2001). In addition, recombinant Mac-1-mediated proteolytic cleavage of the hinge region of IgG is hypothesized to prevent the recognition of antibody-opsonized GAS by Fc receptors of immune cells and by the complement system (von Pawel-Rammingen *et al.*, 2002). During studies utilizing recombinant Mac-1 incubated with serum containing anti-GAS antibodies, subsequent proteolytic degradation inhibited ROS production by human neutrophils (Lei *et al.*, 2001; Söderberg and von Pawel-Rammingen 2008). However, a study comparing a Mac-1-deficient mutant GAS strain to the wild-type and complemented strains demonstrated that native Mac-1 expressed by live GAS does not reduce ROS production or other neutrophil functions (Okumura *et al.*, 2013).

### Mac-2

A second Mac-1-like protein has been identified, designated Mac-2 (Lei *et al.*, 2002a). Previously, Mac-2 has been reported to interfere with opsonophagocytosis by blocking Fcγ receptors on phagocytic cells (Agniswamy *et al.*, 2004). In initial studies, Mac-2 did not inhibit ROS production by neutrophils stimulated with IgG-coated latex beads (Lei *et al.*, 2002a). Subsequent studies measuring immunocomplex-induced oxidative burst in whole blood containing recombinant Mac-2 indicated that Mac-2 contributes to the inhibition of opsonophagocytosis-induced ROS production *ex vivo*. However, this inhibition did not enhance streptococcal survival in bactericidal assays (Söderberg, Engström and von Pawel-Rammingen 2008).

## ENZYMATIC DETOXIFICATION MECHANISMS

**Figure 2.**
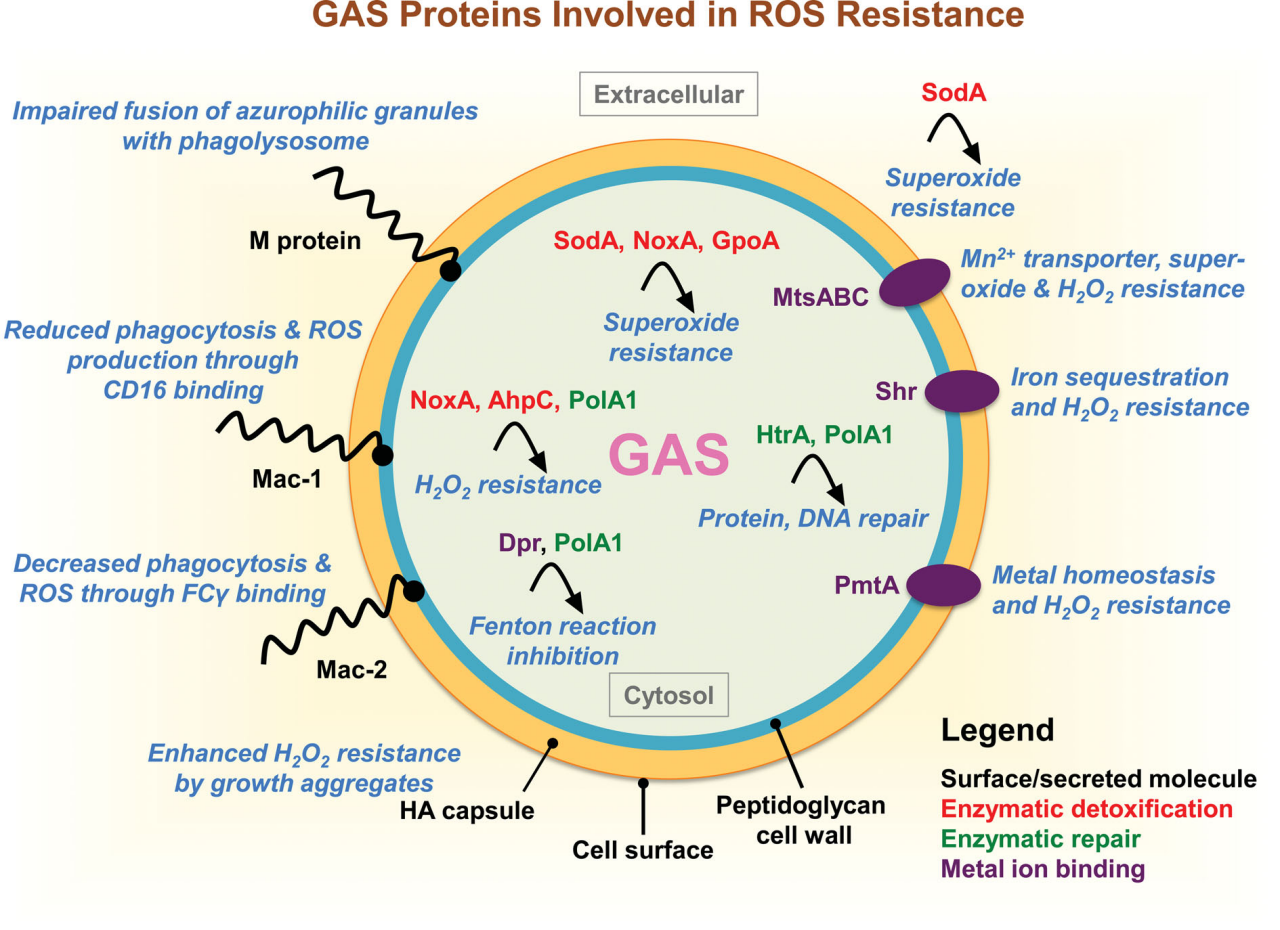
The GAS proteins involved in oxidative stress resistance. Several surface-associated GAS molecules play a role in ROS suppression: M protein impairs azurophilic granule fusion with the phagolysosome in host neutrophils; HA capsular polysaccharide promotes bacterial clumping and enhances H_2_O_2_ resistance; recombinant Mac-1/IdeS binds CD16 on neutrophils inhibiting phagocytosis and ROS production; and Mac-2 binds neutrophil FCγ receptors to inhibit phagocytosis and ROS production. Enzymes involved in superoxide detoxification include NoxA/NOXase, SodA and AhpC. GpoA plays a role in cellular redox homeostasis and protects cells from the deleterious effects of ROS. Chaperone protein HtrA/DegP and DNA polymerase PolA1 function to repair protein or DNA damaged by ROS, respectively. Cation homeostasis, important for oxidative stress resistance, is regulated by several GAS proteins: PmtA, an iron efflux system important for H_2_O_2_ resistance; MtsABC, involved Fe^3+^, Zn^2+^ and Mn^2+^ transport and required for enhanced resistance to superoxide and H_2_O_2_; Shr, a surface-associated heme receptor involved in iron sequestration and H_2_O_2_ resistance; and Dpr/MrgA, plays a pivotal role in resistance to oxidant stress by functioning as an iron (Fe^2+^) chelator and preventing the production of bactericidal hydroxyl radicals via the Fenton reaction. Abbreviations: ROS, reactive oxygen species; HA, hyaluronan; Mac-1/IdeS, Mac-1-like protein; NoxA/NOXase, NADH oxidase A; SodA, superoxide dismutase; AhpC, alkyl hydroperoxidase reductase C; GpoA, glutothione peroxidase; HtrA/DegP, high-temperature requirement A; PolA1, DNA polymerase I; PmtA, PerR-regulated metal transporter A; MtsABC, metal transporter of *Streptococcus* ABC; Shr, streptococcal hemoprotein receptor; Dpr/MrgA, Dps-like peroxide resistance protein.

GAS is a facultative anaerobic organism that generates energy from glycolysis due to a deficiency in heme-containing protein complexes for oxidative phosphorylation. The growth kinetics of this aerotolerant pathogen are substantially enhanced in the presence of oxygen (Gabbianelli *et al.*, 1998). GAS is a member of the lactic acid bacteria family and naturally lacks catalase, a heme-containing peroxidase and highly efficient H_2_O_2_-detoxification enzyme expressed by numerous other bacterial species to survive in aerobic environments. Nevertheless, GAS persists in oxygen-rich anatomical sites of the human host, and has evolved effective strategies to combat bactericidal H_2_O_2_ and other ROS generated by host innate immune cells, such as neutrophils and macrophages, at the site of infection (Gibson *et al.*, 2000). The GAS genome harbors peroxidases, Sod and NADH oxidase to directly decompose ROS (Gibson and Caparon 1996; Gerlach, Reichardt and Vettermann 1998; Gibson *et al.*, 2000; King, Horenstein and Caparon 2000). Indirect ROS resistance mechanisms comprise proteins involved in the repair of ROS-damaged biomolecules (e.g. DNA or proteins) or metal ion transporters involved in maintaining metal homeostasis and oxidative stress resistance (Table 1). Next, we highlight conserved ROS defense mechanisms employed by multiple serotypes of GAS (Fig. 2).

### Superoxide dismutase A (SodA)

Sod represents the first line of bacterial defense against superoxide stress by converting O_2_•^−^ into H_2_O_2_ and O_2_, thereby protecting cells from the toxic effects of superoxide (Fridovich 1997; Liochev and Fridovich 2007). Sods are distinguished by their metal cofactors, which are ferrous (Fe^3+^), manganese (Mn^2+^), nickel (Ni^2+^) or copper/zinc (Cu^2+^/Zn^2+^). *Escherichia coli* has three Sods cofactored with Cu^2+^/Zn^2+^ (periplasm), Mn^2+^ or Fe^3+^ (both cytoplasmic). GAS Sods are highly conserved among different serotypes as well as highly homologous to Sods from other Gram-positive bacteria (Gerlach, Reichardt and Vettermann 1998; Poyart *et al.*, 1998). GAS has a single Mn^2+^-dependent superoxide dismutase, designated SodA, which enhances GAS growth under aerobic conditions (Gibson and Caparon 1996). The H_2_O_2_ generated by SodA is neutralized by endogenous GAS peroxidases (Gerlach, Reichardt and Vettermann 1998). A *sodA* mutant in serotype M14 GAS strain HSC5 (Port, Paluscio and Caparon 2013) is more susceptible to superoxide challenge (Gibson and Caparon 1996). *E. coli* mutants deficient in the periplasmic Cu^2+^/Zn^2+^ Sod are more susceptible to H_2_O_2_, but exhibit equivalent sensitivity towards superoxide challenge (Gort, Ferber and Imlay 1999). Additionally, heterologous expression of SodA from *Streptococcus thermophilus* strain AO45 in four otherwise SodA-negative lactobacilli promoted bacterial survival after challenge with various concentrations of H_2_O_2_ up to 1.6 mM. Treatment of *Lactobacillus gasseri* engineered to express SodA in the presence of an iron chelator, 2′-2′-dipyridyl (DIP) was protective following lethal challenge with 2.5 mM H_2_O_2_. This phenotype was attributed to inhibition of the Fenton reaction, which is downstream of H_2_O_2_ formation (Bruno-Barcena *et al.*, 2004). Extracellular SodA derived from various GAS clinical isolates and reference strains failed to provoke an efficient immune response (McMillan *et al.*, 2004); however, secretion of SodA from serotype M12 GAS strain 12714 into the extracellular milieu protects against the extracellular ROS produced by innate immune cells (Fig. 2) (Gerlach, Reichardt and Vettermann 1998). SodA might be indirectly regulated by the peroxide response regulator (PerR) in GAS as PerR does not bind to the SodA promoter directly, but *perR* mutants in serotype M1 GAS strain AP1 have lower transcript levels of SodA (Ricci, Janulczyk and Bjorck 2002). In response to superoxide, both SodA and the cell surface adhesin fibronectin-binding protein F (PrtF, also known as Sfb) are transcriptionally up-regulated (Gibson *et al.*, 1995; Gibson and Caparon 1996). Furthermore, PrtF is transcriptionally regulated in response to oxygen concentration (VanHeyningen *et al.*, 1993).

### Alkyl hydroperoxide reductase (AhpC)

Using a genome-wide screen for peroxidase-related genes in GAS, King and colleagues identified two genes, alkyl hydroperoxide reductase (*ahpC*) and glutathione peroxidase (*gpoA*), which inactivate inorganic and organic peroxides and thereby enhance GAS resistance to ROS (King, Horenstein and Caparon 2000; Brenot, King and Caparon 2005). Alkyl hydroperoxide reductase (AhpC) catalyzes the pyridine nucleotide-dependent reduction of organic hydroperoxides and H_2_O_2_ (Poole and Ellis 1996). AhpC is present in GAS and closely related streptococci, including *S. mutans* and *S. agalactiae* (group B *Streptococcus*); however, AhpC is absent in *S. pneumoniae* (pneumococcus) and Gram-negative bacteria such as *E. coli* (Yesilkaya *et al.*, 2013). GAS AhpC is an NADH-dependent H_2_O_2_-degrading peroxidase that is directly up-regulated in response to oxidative stress, but is not directly regulated by transcription factor PerR (Brenot, King and Caparon 2005), a negative regulator of the inducible peroxide resistance response in GAS. Compared to wild-type serotype M14 GAS strain HSC5, an isogenic in-frame *ahpC* deletion mutant lacking the central 45 amino acids adjacent to the putative active site residue was more susceptible to a 5–10 mM range of paraquat (methyl viologen), a redox-cycling agent that increases intracellular levels of superoxide; however, the mutation had no effect on growth rate kinetics under aerobic conditions, SodA expression levels or bacterial survival following exposure to 1 mM paraquat (Hassan and Fridovich 1979; King, Horenstein and Caparon 2000). GAS *ahpC* mutants were more susceptible than wild-type to 5–20% cumene hydroperoxide as measured by zone of growth inhibition in disk diffusion assays (King, Horenstein and Caparon 2000). Catalase treatment rescued the *ahpC* mutant phenotype, indicating that AhpC enhances GAS resistance to H_2_O_2_; however, following challenge with millimolar concentrations of H_2_O_2_, no significant decrease in H_2_O_2_ resistance was observed between the *ahpC* mutant and the parental strain (King, Horenstein and Caparon 2000). Interestingly, an *ahp* mutant in a catalase-deficient *E. coli* mutant accumulated more H_2_O_2_ intracellularly indicating a primary role for Ahp in scavenging intracellular H_2_O_2_ (Seaver and Imlay 2001). Recent observations indicate that bacteria are exposed to 5–10 μM H_2_O_2_ in neutrophil phagosomes (Mishra and Imlay 2012), concentrations where peroxidases including AhpC are more kinetically efficient scavengers of H_2_O_2_ than catalase. However, the enzymatic activity of AhpC is limited by NADH availability and AhpC becomes saturated at H_2_O_2_ concentrations around 20 μM (Mishra and Imlay 2012; Imlay 2013). These findings indicate that AhpC from serotype M14 GAS strain HSC5 is not critical for survival in *in vitro* studies using super-physiological concentrations of 4 mM H_2_O_2_, where AhpC is saturated (King, Horenstein and Caparon 2000; Brenot, King and Caparon 2005), but is required for full virulence in a mouse model of soft-tissue infection *in vivo* (Brenot, King and Caparon 2005).

### Glutathione peroxidase (GpoA)

Glutathione peroxidase (Gpo) is a selenoprotein oxidoreductase important for maintaining cellular redox homeostasis and for protecting cells from the deleterious effects of ROS (Arthur 2000). Initial findings using an in-frame *gopA* mutant indicated that GpoA in serotype M14 GAS strain HSC5 is important for resistance against 5–10 mM paraquat (King, Horenstein and Caparon 2000). Even though GpoA was not involved in resistance against direct challenge with H_2_O_2_, the *gpoA* mutant was rescued by addition of catalase, which detoxifies H_2_O_2_, indicating that GpoA also contributes to resistance against downstream products of superoxide (King, Horenstein and Caparon 2000). Subsequent studies with an in-frame *gpoA* mutant in GAS strain HSC5 showed that GpoA contributes to GAS virulence in *in vivo* models of disease characterized by acute inflammation, a condition marked by high ROS levels (Mittal *et al.*, 2014), including subcutaneous and systemic infection models (Brenot *et al.*, 2004). However, GpoA was not essential for virulence in a zebrafish (*Danio rerio*) model of streptococcal myositis, a disease characterized by the absence of inflammatory cell infiltrate (Brenot *et al.*, 2004). These findings suggest that GpoA is essential for GAS to adapt to oxidative stress and indicates that high, non-physiological concentrations of ROS tested in earlier *in vitro* studies may have neglected the important role played by GpoA *in vivo* (King, Horenstein and Caparon 2000; Brenot *et al.*, 2004).

### NADH oxidase A (NoxA)

The NADH oxidase (NoxA or NOXase) expressed by GAS is an important enzyme involved in H_2_O_2_ decomposition and the regeneration of NAD^+^, which plays a pivotal role in several pathways including DNA repair, post-translational protein modifications and apoptosis (Massudi *et al.*, 2012). Single crossover *noxA* mutants in serotype M6 GAS strain JRS4 and serotype M14 GAS strain HSC5 showed reduced growth under high O_2_ conditions and after challenge with paraquat (Gibson *et al.*, 2000). In addition, NoxA-deficient mutants accumulated almost three times more H_2_O_2_ in culture supernatant compared to parental strains. All *noxA* mutant phenotypes were rescued by the addition of catalase to the growth medium (Gibson *et al.*, 2000). Nox enzymes are generally described as a peroxide-resistance mechanism employed by catalase-negative bacteria including GAS. Accordingly, heterologous expression of Nox from *Enterococcus faecalis* in the *noxA* GAS mutants reduced H_2_O_2_ accumulation in culture supernatant (Gibson *et al.*, 2000). A study using purified recombinant Nox revealed that NoxA from GAS is the most potent enzyme among all tested NADH oxidases from multiple bacteria (Gibson *et al.*, 2000; Gao *et al.*, 2012). This finding indicates that GAS has optimized catalase-independent mechanisms to detoxify H_2_O_2_ and that NoxA is an important pathway for GAS to tolerate high O_2_ environments and promote resistance to ROS (Gibson *et al.*, 2000; Gao *et al.*, 2012).

## ENZYMATIC REPAIR MECHANISMS

A sub-lethal dose of ROS damages proteins, DNA and has multiple adverse effects on the bacterium. Therefore, an immediate repair of the damage is essential for the survival of the bacterium. GAS is equipped with efficient repair mechanisms (Table 1). However, this rather indirect contribution to resistance to oxidative stress is proportionally difficult to determine. In the following section, we focus on several key proteins contributing to the repair of ROS-induced cell damage (Fig. 2).

### High-temperature requirement A (HtrA)

High-temperature requirement A (HtrA, also designated DegP) is a dual-functional serine protease and chaperone protein that either refolds or degrades damaged proteins destined for secretion into the extracellular environment (Spiess, Beil and Ehrmann 1999). Homologs of this protein are present in many Gram-negative bacteria including *E. coli* (Bringer *et al.*, 2005) and Gram-positive bacteria such as *S. aureus* (Rigoulay *et al.*, 2005). HtrA in serotype M14 GAS strain HSC5 is involved in secretion of virulence factors via the ExPortal, a discrete cytoplasmic membrane microdomain involved in the biogenesis of secreted GAS proteins (Rosch and Caparon 2005). HtrA indirectly affects the maturation kinetics of the secreted cysteine protease streptococcal pyrogenic exotoxin B (SpeB), with the absence of a functional HtrA causing a delay in the biogenesis of active SpeB protease (Cole *et al.*, 2007). Studies with an insertional *htrA* mutant in serotype M6 GAS strain S43 (ATCC 12348) showed a growth defect at 37 and 44°C, an inhibition zone around disks with 200 mM paraquat, and HtrA was necessary for full virulence in a mouse model of infection (Jones *et al.*, 2001). A more recent study using an insertional *htrA* mutant in GAS confirmed the earlier report showing a growth defect at 37°C (Jones *et al.*, 2001; Lyon and Caparon 2004); however, an in-frame *htrA* mutant in serotype M14 GAS strain HSC5 had no growth defect at 37°C (Lyon and Caparon 2004). Therefore, it remains unclear if the defect in oxidative stress resistance observed with *htrA* mutant in GAS strain S43 might be due to secondary effects rather than the direct activity of HtrA.

### DNA polymerase I (PolA1)

Recently, a DNA polymerase I (PolA1) was identified in GAS, expressed from the same five-gene operon as the peroxide stress response regulator PerR (Toukoki and Gryllos 2013). An in-frame *polA1* mutant in a serotype M3 GAS strain 003Sm was hypersensitive to challenge with 10 mM H_2_O_2_ compared to the wild-type and complemented strains. In addition, the mutant was rescued after challenge with H_2_O_2_ in the presence of an iron chelator deferoxamine mesylate (desferal, DFM) or the hydroxyl radical scavenger thiourea suggesting that the hypersensitivity of the mutant occurred via the Fenton reaction (Toukoki and Gryllos 2013). Adaptation with sub-lethal H_2_O_2_ prior to H_2_O_2_ lethal challenge decreased the killing of the double *perR polA1* mutant 9-fold indicating that PolA1 is involved in a PerR-dependent oxidative stress defense (Toukoki and Gryllos 2013). Furthermore, the *polA1* mutant exhibited a reduction in the repair of DNA damage initiated by UV light or ciprofloxacin (Toukoki and Gryllos 2013), a fluoroquinolone antibiotic that induces double-strand DNA breaks via entrapment of topoisomerases during DNA cleavage (Chen *et al.*, 1996). PolA1 also contains a 5′–3′ exonuclease domain and increased mutation rates associated with PolA1 function to promote genetic diversity (Toukoki and Gryllos 2013).

## ROLE OF CATIONS IN GAS RESISTANCE TO OXIDATIVE STRESS

The acquisition of metal ions such as iron (Fe), manganese (Mn), cobalt (Co) and zinc (Zn) is important for the survival of bacterial pathogens inside the human host. Metal ions enhance oxidative stress resistance by directly detoxifying ROS, serving as cofactors for enzymes such as SodA, and acting as signaling molecules for the transcriptional regulation of genes involved in ROS defense (Yesilkaya *et al.*, 2013). However, high intracellular concentrations of metal ions may be bactericidal or induce oxidative stress (Finney and O'Halloran 2003); therefore, GAS has evolved effective systems to tightly regulate metal ion homeostasis (Nelson 1999). A recent study in serotype M1T1 GAS strain 5448, a representative of the globally disseminated serotype M1T1 clone (Cole *et al.*, 2011), demonstrated that mutants in a putative Zn^2+^ efflux/activator system, *czcD* (Spy_0653) and *gzcA* (Spy_0654) resulted in increased susceptibility to Zn^2+^ and clearance by innate immune cells *in vitro* and in a mouse model of soft-tissue infection (Ong *et al.*, 2014). These findings indicate that Zn^2+^ contributes to clearance of bacterial pathogens by acting as an antimicrobial factor, and that Zn^2+^ efflux systems are important for full GAS virulence.

Neutrophils are the first line of defense against invading pathogens. Calprotectin from neutrophils binds and reduces the availability of Mn^2+^ and Zn^2+^ at the site of infection, which decreases the activity of the Mn^2+^-dependent Sod in *S. aureus* (Kehl-Fie *et al.*, 2011). Inhibition of Sod results in a higher susceptibility of *S. aureus* by promoting clearance of innate immune cells *in vitro* and *in vivo* (Kehl-Fie *et al.*, 2011). MtsABC from GAS functions as a Mn^2+^ transporter and GAS deficient in MtsABC are more susceptible to superoxide probably due to the decreased activity of SodA (Janulczyk, Ricci and Bjorck 2003), as described in more detail below. In addition, GAS requires low levels of Fe^3+^ for growth and survival in the human host; however, GAS preferably utilizes Mn^2+^ as cofactor for enzymes possibly to minimize iron-induced radical formation via the Fenton reaction as described for *S. pneumoniae* (Ong *et al.*, 2013). GAS has multiple metal transporters and receptors. Herein, we focus on the contribution of metal transport systems to ROS resistance (Fig. 2).

### PerR-regulated metal transporter A (PmtA)

In a genome-wide transcriptome analysis between serotype M14 GAS strain HSC5 and an in-frame *perR* mutant in the absence of a stress stimulus, six genes were identified with at least 3-fold differential expression (Brenot, Weston and Caparon 2007). Among those genes, five were up-regulated and one was down-regulated. One of the up-regulated genes in the *perR* mutant is the PerR-regulated metal transporter A (PmtA), which has a PerR-binding site and is a putative iron efflux protein in GAS. Overexpression of PmtA in the *perR* mutant is responsible for a higher resistance of up to 1.75 mM zinc, concentrations to which the wild-type and *pmtA* mutant were susceptible. In addition, an in-frame *pmtA* mutant was more sensitive to challenge with 4 mM H_2_O_2_ compared to the wild-type. However, in an *in vivo* mouse model of soft-tissue infection, *pmtA* was not found to be up-regulated compared to mid-log growth phase (Brenot, Weston and Caparon 2007). Therefore, the exact role of PmtA *in vivo* remains unclear. A similar global transcriptional analysis in serotype M3 GAS strain 003Sm confirmed that *pmtA* is highly regulated by PerR. Interestingly, PerR-regulated genes were essential for GAS pharyngeal colonization and ROS-dependent phagocyte resistance indicating an important role for PmtA in physiology and potentially virulence (Gryllos *et al.*, 2008).

### Dps-like peroxide resistance protein (Dpr)

In the presence of iron, H_2_O_2_ is readily converted to the highly reactive OH• through the Fenton reaction. The Dps-like peroxide resistance protein (Dpr, also designated MrgA) plays a pivotal role in resistance to oxidant stress by functioning as a chelator of intracellular iron (Fe^2+^) to prevent the production of bactericidal hydroxyl radicals via the Fenton reaction (Andrews, Robinson and Rodriguez-Quinones 2003; Tsou *et al.*, 2008; Haikarainen *et al.*, 2010; Ge and Sun 2014). Dpr has also been reported to protect GAS from pH-induced stress (Tsou *et al.*, 2008), and is transcriptionally repressed by PerR (Brenot, King and Caparon 2005; Tsou *et al.*, 2010), as discussed below. A study using an in-frame allelic exchange *dpr* mutant in serotype M1 GAS strain A-20 found that the mutant was hypersensitive to killing by 5 mM H_2_O_2_, compared to wild-type and complemented strains (Tsou *et al.*, 2008). Addition of the iron chelator DFM leads to a dose-dependent increase in survival of the mutant strain following *in vitro* challenge with 5 mM H_2_O_2_ (Tsou *et al.*, 2008). Similarly, an in-frame *dpr* mutant in serotype M14 GAS strain HSC5 did not exhibit a defect in growth under aerobic conditions, nor the ability to degrade peroxide, and was hypersensitive to high concentrations of H_2_O_2_, compared to wild-type (Brenot, King and Caparon 2005). These data indicate that Dpr functions as an iron chelator by reducing free iron and thereby increasing the fitness of GAS inside the human host. In *Bacillus subtilis* and *S. aureus* (Chen and Helmann 1995), MrgA plays a central role in protection from a lethal challenge of peroxide. In *S. mutans*, Dpr is important for growth under aerobic conditions (Yamamoto *et al.*, 2000). The transcriptional regulator PerR directly regulates the expression of Dpr in GAS (Brenot, King and Caparon 2005).

### Metal transporter of *Streptococcus* ABC (MtsABC/SiaABC)

In order to establish an infection and survive within the host, many Gram-positive bacterial pathogens utilize ATP-binding cassette (ABC) transporters to acquire essential nutrients and metal ions such as copper, manganese, iron, zinc and cobalt (Claverys 2001; Higgins 2001). Metal homeostasis plays an important role in GAS resistance to oxidative stress and virulence (Ge and Sun 2014), and three metal ABC transporters have been described so far: 1) MtsABC is involved in the uptake and transport of a variety of cations in GAS (Fe^3+^, Zn^2+^ and Mn^2+^) (Janulczyk, Pallon and Bjorck 1999; Janulczyk, Ricci and Bjorck 2003); 2) SiaABC/HtsABC transports heme (Payne 1993); and 3) FtsABC is the principal transporter of Fe^3+^ and heme (Hanks *et al.*, 2005; Ge and Sun 2014). Compared to the wild-type strain, a *perR* mutant exhibited a reduction in *mtsA* transcript levels suggesting that PerR up-regulates *mtsABC* transcription (Ricci, Janulczyk and Bjorck 2002). MtsABC-deficient GAS exhibit reduced growth rates in metal-depleted medium and under aerobic conditions (Janulczyk, Ricci and Bjorck 2003). The *mtsABC* mutant in serotype M1 GAS strain AP1 was hypersensitive to killing by 5 mM H_2_O_2_ and paraquat-induced superoxide radicals following growth in the presence of 2 or 10 mM paraquat, in comparison to wild-type (Janulczyk, Ricci and Bjorck 2003). In GAS, Mn-dependent SodA plays an important role in bacterial resistance to oxidative stress (Gibson and Caparon 1996; Fridovich 1997; Ferretti *et al.*, 2001). The reduced SodA enzymatic activity of the *mtsABC* mutant, most likely due to low intracellular levels of manganese resulting from defective manganese transport, may account for this defect in ROS resistance. Supplementation of the culture medium with 30 μM MnCl_2_ restored SodA enzymatic activity and growth rate to wild-type levels in the presence of paraquat. The *mtsABC* mutant strain was ∼30-fold less virulent compared to wild-type in an *in vivo* mouse air sac model of subcutaneous infection. These data demonstrate that metal cation acquisition by MtsABC plays a key role in GAS growth, oxidative stress resistance and virulence (Janulczyk, Ricci and Bjorck 2003). Additionally, MtsABC shares homology with the pneumococcal surface antigen A, the lipoprotein part of an ABC Mn^2+^ transporter, which also functions as an adhesin and is necessary for full virulence in a mouse model of soft-tissue infection (Berry and Paton 1996).

### Streptococcal hemoprotein receptor (Shr)

Iron availability is essential for bacterial growth, survival and the establishment of infection within the human host (Payne 1993). Streptococcal hemoprotein receptor (Shr) is the first protein of the streptococcal iron acquisition (Sia) operon and is necessary for maintenance of iron homeostasis (Bates *et al.*, 2003). Shr is a surface-associated and secreted receptor for heme, which is the main source of iron in human blood (Bates *et al.*, 2003; Fisher *et al.*, 2008). Insertional inactivation of the *shr* gene in serotype M1 GAS strain SF370 enhanced bacterial survival after challenge with 5 mM H_2_O_2_, compared to wild-type. However, the addition of hemoglobin increased the survival of wild-type to the same level of the *shr* mutant, suggesting that Shr is involved in hemoglobin-dependent resistance to H_2_O_2_ (Bates *et al.*, 2003). A recent study demonstrated that Shr is important for survival of serotype M1T1 GAS strain 5448 in human whole blood and full virulence in two *in vivo* mouse models of infection, underscoring the important role for metal cations in GAS pathogenesis (Dahesh, Nizet and Cole 2012). These studies reveal that during bloodstream infections, hemoglobin sequestration by Shr is an important defense mechanism of GAS to survive inside the human host, presumably by capturing free Fe^2+^ and thereby preventing the production of highly bactericidal hydroxyl radicals.

## GAS REGULATION OF THE OXIDATIVE STRESS RESPONSE

Stress response plays an important role in the regulation of virulence and gene expression in bacterial pathogens; however, the σ^B^ general stress response pathway involved in the regulation of stress-induced genes in many Gram-positive species (Volker *et al.*, 1994) is absent in GAS. A coordinated response to oxidative stress is necessary for GAS to establish an infection and cause disease. Several two-component systems or stand-alone transcriptional regulators respond to extracellular stimuli and have been linked to the GAS oxidative stress response, including PerR, Rgg/RopB, Ihk-Irr, MtsR and CiaRH.

### PerR regulon

**Figure 3.**
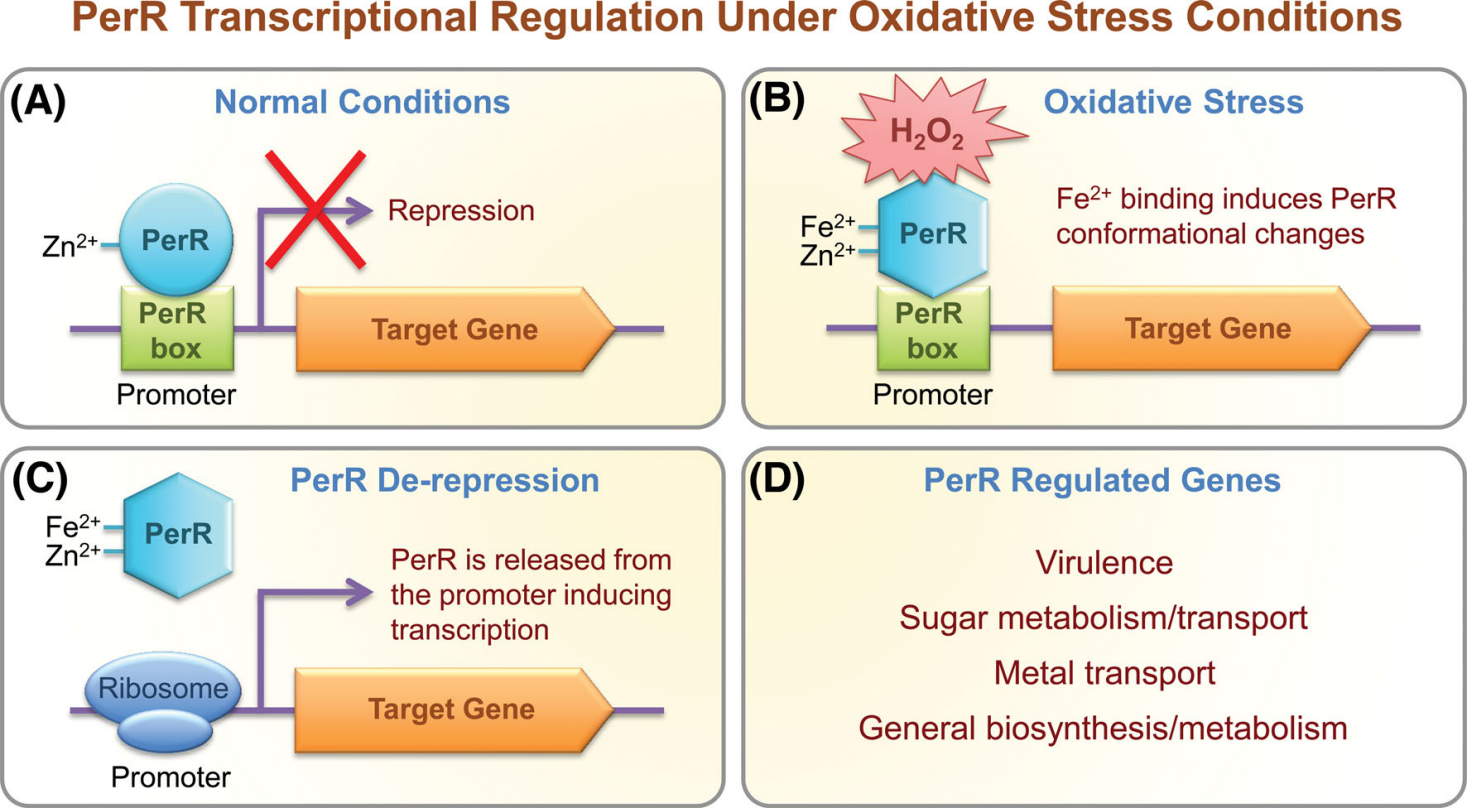
Transcriptional regulator PerR regulates the inducible peroxide resistance response. **(A)** Under normal conditions, PerR has a bound zinc ion (Zn^2+^) and generally represses transcription of PerR-regulated genes by directly binding conserved sequences, known as Per boxes, in the promoter of each target gene. **(B)** Under conditions of peroxide stress (H_2_O_2_), a ferrous ion (Fe^2+^) is bound, resulting in the oxidation of histidine residues and conformational changes within PerR. **(C)** Conformational changes reduce the DNA-binding affinity of PerR, resulting in the dissociation of PerR from the PerR boxes, and transcription of the target gene. **(D)** GAS PerR regulates genes involved in virulence, sugar metabolism/transport, metal ion efflux and housekeeping metabolic pathways.

Regulation of the inducible peroxide resistance response in GAS is primarily coordinated by the peroxide-sensing PerR transcriptional regulator, a 155 amino acid zinc-containing metalloprotein, which is a member of the ferric uptake regulator (Fur) family of metal-binding transcriptional regulators (Herbig and Helmann 2001; Mongkolsuk and Helmann 2002; Brenot, King and Caparon 2005; Moore and Helmann 2005). PerR is a negative transcriptional regulator, or repressor, of the inducible peroxide resistance response in GAS (King, Horenstein and Caparon 2000; Ricci, Janulczyk and Bjorck 2002), and contributes to GAS iron homeostasis, oxidative stress response and virulence (Ricci, Janulczyk and Bjorck 2002; Brenot, King and Caparon 2005; Gryllos *et al.*, 2008) (Table 2). PerR generally acts as a repressor by directly binding the promoter of the target gene (Mongkolsuk and Helmann 2002; Hayashi *et al.*, 2005). Peroxide sensing by PerR requires regulatory metal ions, and under oxidative stress conditions PerR conformational changes induced by the oxidation of metal ions reduce the DNA-binding affinity of PerR, de-repressing target gene expression (Dubbs and Mongkolsuk 2012) (Fig. 3). The PerR of *B. subtilis* has been well characterized and shown to repress the expression of target genes by binding to conserved promoter sequences known as Per boxes (Chen, Keramati and Helmann 1995). However, with the exception of *pmtA* and *ahpCF*, the Per box sequence is not fully conserved in promoters regulated by PerR in GAS (Brenot, King and Caparon 2005; Brenot, Weston and Caparon 2007). In Gram-positive species such as *B. subtilis* and *S. aureus*, PerR coordinately controls the oxidative stress response genes and iron homeostasis (Chen, Keramati and Helmann 1995; Horsburgh *et al.*, 2001; Helmann *et al.*, 2003) in order to prevent the Fenton reaction, whereby intracellular iron reacts with H_2_O_2_ to form highly toxic and oxidizing hydroxyl radicals (OH•).

**Table 2.** PerR-regulated genes identified by microarray analyses and confirmed by quantitative real-time PCR (qRT-PCR) for different GAS serotypes.

M type	ORF no.^a^	Growth phase	Gene	Protein^b^	Relative expression^c^		Reference^d^
					Microarray	qRT-PCR	
*Oxidative stress – virulence (putative)*							
M3	0466	Mid-exponential	*adcA*	Putative adhesin (zinc-binding)	−4.6	−3.2	Gryllos *et al.* (2008)
M3	0815	Mid-exponential	*hylIII*	Putative hemolysin III	+3.0	−1.2	Gryllos *et al.* (2008)
M3	1093	Mid-exponential	*pmtA*	Putative metal transport ATPase	−11.3	−15.8	Gryllos *et al.* (2008)
M14	05800	Mid-exponential	*pmtA*	Putative metal transport ATPase	n/a	−30.0	Brenot, Weston and Caparon (2007)
M3	1770	Late exponential	*ahpC*	Putative alkyl hydroperoxidase	−1.7	−1.9	Gryllos *et al.* (2008)
M14	08790	Mid-exponential	*ahpC*	Putative alkyl hydroperoxidase	n/a	−1.3	Brenot, King and Caparon (2005)
M3	1095	Late exponential	*mf4*	Putative mitogenic factor/DNase	−2.2	−2.1	Gryllos *et al.* (2008)
M1	1436	Mid-exponential	*mf3*	Secreted DNase virulence factor	n/a	+4.3	Wen *et al.* (2011)
M3	0298	Late exponential	*prtS*	Interleukin 8 protease	−2.1	−2.0	Grifantini *et al.* (2011)
M3	0482	Late exponential	*sagC*	SLS-associated ORF	−2.1	−1.6	Grifantini *et al.* (2011)
M14	06285	Mid-exponential	*mrgA*	Peroxide resistance protein	n/a	−2.8	Brenot, King and Caparon (2005)
M14	08560	Mid-exponential	*lsp*	Laminin-binding protein	n/a	−100	Brenot, Weston and Caparon (2007)
M1 T1	1415	Mid-exponential	*sda1*	DNase and virulence factor	n/a	+2.5	Wang *et al.* (2013)
*Sugar metabolism/transport*							
M3	1489	Late exponential	*lacR.1*	Putative lactose PTS repressor	+1.9	+2.5	Gryllos *et al.* (2008)
M3	1484	Mid-exponential	*lacB.1*	Galactose 6-phosphate isomerase	−2.3	+1.1	Gryllos *et al.* (2008)
M3	1658	Late exponential	*lacB.2*	Galactose 6-phosphate isomerase	+5.0	+6.6	Gryllos *et al.* (2008)
M3	1658	Late exponential	*lacB.2*	Galactose-6-phosphate isomerase	−4.9	−2.0	Grifantini *et al.* (2011)
M3	1654	Late exponential	*lacE*	Putative PTS enzyme IIBC	+4.0	+10.7	Gryllos *et al.* (2008)
M3	1659	Late exponential	*lacA2*	Galactose-6-phosphate isomerase	−2.4	−2.5	Grifantini *et al.* (2011)
M3	1660	Late exponential	*lacR.2*	Putative lactose PTS repressor	+1.9	+2.2	Gryllos *et al.* (2008)
M3	1487	Late exponential	*pts*	Putative PTS enzyme IIB	+2.9	+5.9	Gryllos *et al.* (2008)
*Metal – ABC transport*							
M3	0069	Mid-exponential	*adcR*	Putative transcriptional repressor	−1.9	−1.3	Gryllos *et al.* (2008)
M3	0069	Mid-exponential	*adcR*	Putative transcriptional repressor	+1.6	+2.3	Grifantini *et al.* (2011)
M3	0071	Mid-exponential	*adcB*	Zinc/manganese ABC transporter	+2.0	+1.7	Grifantini *et al.* (2011)
M3	0319	Mid-exponential	*mtsB*	Iron ABC transporter	−1.9	−1.0	Grifantini *et al.* (2011)
M3	1557	Late exponential	*siaD*	Putative ABC transporter	−1.9	−1.7	Grifantini *et al.* (2011)
M3	1560	Late exponential	*shp*	Heme/ferrichrome-binding protein	−2.0	−1.8	Grifantini *et al.* (2011)
M1	0453	Mid-exponential	*mtsA*	Iron ABC transporter	n/a	−2.0	Hanks *et al.* (2006)
*General biosynthesis/metabolism*							
M3	0027	Mid-exponential	*purE*	Phosphoribosylaminoimidazole carboxylase catalytic subunit	+2.2	+1.2	Gryllos *et al.* (2008)
M3	1615	Mid-exponential	*rpsN*.2	30S subunit ribosomal protein S14	−3.3	−2.5	Gryllos *et al.* (2008)
M14	07960	Mid-exponential	*rpsN*.2	30S subunit ribosomal protein S14	n/a	−4.0	Brenot, Weston and Caparon (2007)
M3	0302	Late exponential	*nrdI.2*	Putative ribonucleotide reductase	−2.7	−1.5	Gryllos *et al.* (2008)
M3	0013	Late exponential	n/a	Putative amino acid permease	−2.5	−1.5	Grifantini *et al.* (2011)
M3	0217	Late exponential	*oppC*	Oligopeptide permease	−2.0	−1.9	Grifantini *et al.* (2011)
M3	1363	Late exponential	*cysM*	Putative *O*-acetylserine lyase	+2.1	1.0	Grifantini *et al.* (2011)
M3	1794	Late exponential	*nrdD*	Ribonucleoside triphosphate reductase	−2.9	−2.5	Grifantini *et al.* (2011)
*Hypothetical*							
M3	1208	Mid-exponential	n/a	Conserved hypothetical protein	+2.0	+2.0	Gryllos *et al.* (2008)
M3	1724	Late exponential	*phtD*	Hypothetical protein	−2.3	−5.6	Gryllos *et al.* (2008)
M14	08555	Mid-exponential	*phtD*	Hypothetical protein	n/a	−100	Brenot, Weston and Caparon (2007)
M14	05535	Mid-exponential	*phtY*	Hypothetical protein	n/a	−30	Brenot, Weston and Caparon (2007)
*Miscellaneous*							
M3	0840	Mid-exponential	n/a	Putative transcriptional repressor	+3.2	+4.6	Grifantini *et al.* (2011)
M14	08775	Mid-exponential	*csp*	Putative cold shock protein	n/a	+2.5	Brenot, King and Caparon (2005)

^a^Open reading frame (ORF) number of published GAS genome sequence: M1 strain SF370 (Ferretti *et al.*, 2001), M1T1 strain MGAS5005 (Sumby *et al.*, 2005), M3 strain MGAS315 (Beres *et al.*, 2002) and M14 strain HSC5 (Port, Paluscio and Caparon 2013).

^b^NCBI annotation.

^c^Fold-change of expression in wild-type GAS compared to expression in the isogenic *perR* mutant.

^d^Gryllos *et al.* (2008): Transcriptome comparisons performed between wild-type M3 GAS strain 003Sm and isogenic *perR* mutant strain 003Sm*perR*Δ grown to mid-exponential phase (OD_600nm_ = 0.25) or late-exponential phase (OD_600nm_ = 0.6) phase.

Grifantini *et al.* (2011): Wild-type M3 GAS strain 003Sm and *perR* mutant 003Sm*perR*Δ were grown to mid-exponential phase (OD_600nm_ = 0.25) or late-exponential phase (OD_600nm_ = 0.6) at which point they were challenged with H_2_O_2_ (final concentration 0.5 mM) for 15 min at 37°C or with water as a control.

Brenot, King and Caparon (2005): Total RNA extracted from mid-exponential phase (OD_600nm_ = 0.3) cultures of wild-type serotype M14 GAS strain HSC5 and the isogenic *perR* mutant.

Brenot, Weston and Caparon (2007): RNA from HSC5 and the isogenic PerR-deficient mutant (HΔPer) was isolated from mid-exponential phase cultures (OD_600nm_ = 0.3).

Wen *et al.* (2011): Transcriptome comparisons were performed between wild-type M1 strain AP-20 and isogenic *perR* mutant strain (SW-612) cultured to mid-log phase (OD_600nm_ ∼ 0.5).

Wang *et al.* (2013): Wild-type serotype M1 GAS strain A-20 and isogenic *perR* mutant (SW-612) were grown for 3 h and treated with 0.5 mM of H_2_O_2_ for another 2 h prior to RNA extraction and real-time PCR analysis.

Hanks *et al.* (2006): Serotype M1 GAS strain MGAS5005 and the isogenic *perR* mutant were harvested at an OD_600nm_ of 0.2, 0.4 or 0.7 and immediately processed to isolate total RNA.

n/a; not available.

### Structure of PerR

The unique 11 amino acid residue N-terminal HXH metal-binding motif of PerR is highly conserved among GAS isolates and plays a role in oxidative stress sensing, metal ion binding and GAS virulence (Makthal *et al.*, 2013). Fluorescence polarization assays indicate that metal ion binding enhances the DNA binding affinity of PerR, but has no influence on the sequence-specific DNA binding. Gel mobility shift assays demonstrate that PerR directly senses peroxide stress conditions through iron-dependent metal-catalyzed oxidation, relieving repression of target genes by dissociating from the binding site in the promoter region (Makthal *et al.*, 2013). The PerR crystal is a homodimer containing two metal-binding sites within the dimerization domain (site 1) and interdomain region (site 2) (Makthal *et al.*, 2013). The zinc-binding at site 1 contributes to structural integrity and PerR dimerization, while the metal-binding at site 2 is essential for peroxide sensing, gene regulation and full virulence in a mouse model of systemic GAS infection (Makthal *et al.*, 2013). Under normal conditions in *B. subtilis*, PerR has a bound zinc ion and represses transcription of PerR-regulated genes by directly binding to the PerR boxes in the promoter region. In the presence of oxidative conditions, a ferrous ion (Fe^2+^) is bound, resulting in the oxidation of three histidines, the release of PerR from the PerR boxes and de-repression of the PerR regulon (Herbig and Helmann 2001; Lee and Helmann 2006) (Fig. 3).

### Transcriptomic and proteomic studies of PerR-deficient GAS

The PerR regulon varies considerably among GAS serotypes. For example, the PerR regulon in serotype M5 GAS consists of 6 genes (Brenot, King and Caparon 2005; Brenot, Weston and Caparon 2007), whereas the M3 GAS PerR regulon contains 42 genes (Gryllos *et al.*, 2008). Importantly, not all studies identify the same PerR-regulated genes, perhaps reflecting differences in growth phase (mid-exponential vs. late-exponential) or serotype-dependent variation among GAS isolates (Table 2).

#### Serotype M1 GAS

Precise allelic replacement mutagenesis of *perR* in serotype M1 GAS strain AP1 was performed by replacing the DNA- and metal-binding domains of *perR* (residues 202 to 361) with a kanamycin resistance gene, and complemented with a plasmid containing a functional *perR* gene and promoter region (Ricci, Janulczyk and Bjorck 2002). Northern blot analysis of the AP1 *perR* mutant cultured to exponential growth phase revealed a 48% reduction in iron (^55^Fe) incorporation from the culture medium, and a 2.4-fold reduction in the transcription of metal-binding ABC transporter *mtsA* (Janulczyk, Pallon and Bjorck 1999), compared to wild-type (Ricci, Janulczyk and Bjorck 2002), suggesting that PerR positively activates *mtsABC* transcription (Ricci, Janulczyk and Bjorck 2002). The *in vitro* growth rate of the AP1 *perR* mutant was similar to wild-type following growth in Todd-Hewitt medium supplemented with 0.2% yeast extract (THY), metal-depleted THY and iron-repleted THY (100 μM ferric citrate) under aerobic conditions. The *perR*-deficient mutant was hyperresistant to H_2_O_2_ stress as assessed by growth of mid-log phase cultures exposed to 5 mM peroxide stress for 30 min. The *perR* mutant was more sensitive to the superoxide anion, as indicated by poor growth in the presence of 10 mM paraquat. Compared to wild-type, the PerR-deficient strain had an ∼3-fold transcriptional reduction of *sodA* suggesting that PerR plays a role in the regulation of SodA expression and resistance to superoxide stress in GAS. High intracellular concentrations of H_2_O_2_ may de-repress ROS-responsive regulation, resulting in the partial repression of SodA to minimize H_2_O_2_ production, and the down-regulation of *mtsABC* to reduce intracellular Fe^2+^ and the production of bactericidal hydroxyl radicals by the Fenton reaction. Compared to the parental A-20 strain, the *perR* mutant was attenuated for virulence in a BALB/c mouse skin air sac infection model of GAS infection, consistent with previous reports (Ricci, Janulczyk and Bjorck 2002; Brenot, King and Caparon 2005; Gryllos *et al.*, 2008). It is unclear whether the attenuation was a consequence of *perR* inactivation, the down-regulation of *sodA* expression or the reduced ability of the *perR* mutant to grow under iron-restricted conditions *in vivo*.

Secreted proteins from wild-type serotype M1 GAS strain A-20 and an isogenic *perR* mutant (SW-612) cultured to stationary phase in conditional medium with a protease inhibitor cocktail were compared using two-dimensional (2D) gel electrophoresis to ascertain how PerR regulates the expression of the GAS secretome (Wen *et al.*, 2011). Twenty-five proteins were down-regulated and 13 proteins were up-regulated in the *perR*-deficient mutant, compared to wild-type (Wen *et al.*, 2011). Approximately 50% of the PerR-regulated proteins identified were predicted to play a role in sugar metabolism and stress response. Activation of sugar metabolic pathways may be necessary for GAS to mobilize the additional energy needed to survive and proliferate under oxidative stress conditions (Wen *et al.*, 2011).

#### Serotype M3 GAS

Mutation of *perR* in M3 GAS strain 003Sm enhanced sensitivity to phagocytic clearance by whole human blood and mouse macrophages (Gryllos *et al.*, 2008). Inhibition of phagocyte oxidative burst with diphenyleneiodonium chloride rescued the *perR* mutant phenotype, suggesting that the PerR regulon enhances GAS resistance to phagocyte oxidative burst. Competitive co-infection experiments with wild-type and *perR*-deficient bacteria demonstrated that the *perR* mutant was more rapidly cleared and attenuated for virulence in a baboon model of GAS pharyngitis. Comparative analysis of global gene expression in wild-type M3 GAS and the isogenic *perR* mutant cultured to late-exponential phase identified 42 genes regulated by the PerR regulon (Gryllos *et al.*, 2008). Approximately 50% of the PerR-dependent genes were predicted to encode proteins involved in oxidative stress resistance, virulence, sugar transport and metabolism, which may reflect the increased energy required for GAS to survive the harsh oxidative conditions generated by the host innate immune response (Gryllos *et al.*, 2008). These data indicate that PerR regulates the expression of a diverse set of genes, enhances GAS resistance to phagocytic clearance and contributes to pharyngeal colonization in a non-human primate model of GAS pharyngeal colonization (Gryllos *et al.*, 2008).

Grifantini and coworkers (Grifantini *et al.*, 2011) conducted a transcriptomic analysis of a *perR* mutant in serotype M3 GAS strain 003Sm cultured in the presence of H_2_O_2_. Analysis of wild-type and *perR* mutant transcriptomes revealed that 76 of 237 peroxide-regulated genes were PerR dependent. The PerR-regulated genes, which encode purine and deoxyribonucleotide biosynthesis enzymes, peptide transport and heme uptake, were mostly down-regulated. 53% of the 161 PerR-independent genes were repressed, and encoded for proteins with similar functions to PerR-regulated genes (Grifantini *et al.*, 2011). The 75 up-regulated genes encoded for proteins involved in the detoxification of ROS, repair of damaged DNA, cofactor metabolism and pilus biosynthesis. The strong activation of metabolic enzymes and DNA damage repair mechanisms may play a key role in GAS survival in oxidative environments *in vivo*, similar to *S. aureus* (Chang *et al.*, 2006; Grifantini *et al.*, 2011; Le Breton *et al.*, 2013; Toukoki and Gryllos 2013). PerR-dependent regulation was restored by complementation of the *perR* mutant with the wild-type PerR protein, but not with a modified PerR containing a mutation in one of the two metal-binding sites (Grifantini *et al.*, 2011). Metal content analysis revealed that PerR binds zinc and iron, and that iron oxidation plays a key role in the PerR response (Grifantini *et al.*, 2011). The binding of PerR to the promoter following treatment with EDTA suggests that iron is not required for DNA binding, but is required for optimal PerR-regulated peroxide responses. Reduced iron/heme uptake and increased Mn^2+^ import may augment the substitution of Fe^2+^ with the Fenton-insensitive Mn^2+^ and permit maximal enzymatic activity under oxidative conditions, as previously described for *E. coli* (Anjem, Varghese and Imlay 2009).

#### Serotype M5 GAS

Comparative transcriptomic analysis of wild-type M5 GAS and an isogenic *perR* mutant cultured to mid-log growth phase in the absence of oxidative stress revealed one highly down-regulated gene (*czcD*), a newly identified GAS virulence factor (Ong *et al.*, 2014) and five highly up-regulated genes in the *perR* mutant (Brenot, Weston and Caparon 2007). Of the five up-regulated genes, only *pmtA*, encoding for PmtA involved in metal homeostasis and transport, contained a PerR-binding site in the promoter region and was directly repressed by PerR (Brenot, Weston and Caparon 2007). The ABC-type metal transporter and transcriptional regulator, AdcR, repressed the expression of the remaining genes (*phtY, phtD, lsp* and *rpsN2*). Mutagenesis of all genes in the *perR* mutant background revealed that only *pmtA* contributes to H_2_O_2_ stress resistance *in vitro* (Brenot, Weston and Caparon 2007). The indirect effect on the remaining genes was attributed to metal ion starvation mediated by AdcR (Brenot, Weston and Caparon 2007). The overexpression of *pmtA* also up-regulated the AdcR-regulated genes, suggesting a link between metal ion homeostasis and the PerR and AdcR oxidative stress responses. Up-regulation of PmtA enhanced the resistance of M5 GAS *perR* mutants to H_2_O_2_ *in vitro* (Brenot, Weston and Caparon 2007). However, PmtA overexpression increased metal efflux and may potentiate metal starvation, which could (at least in part) explain the reduced virulence of *perR* mutants *in vivo*.

#### Serotype M14 GAS

The growth kinetics of a *perR* mutant in serotype M14 GAS strain HSC5 containing an in-frame deletion in the N-terminal DNA-binding region of PerR was similar to wild-type under aerobic conditions (King, Horenstein and Caparon 2000). However, compared to wild-type bacteria, *perR* mutant survival was dramatically enhanced in lethal concentrations of H_2_O_2_ (King, Horenstein and Caparon 2000). De-repression of the transcription of *ahpC, gpoA* and *mrgA* was not observed for the HSC5 *perR* mutant (King, Horenstein and Caparon 2000), in contrast to a *perR* mutant reported for *B. subtilis* (Chen and Helmann 1995; Bsat *et al.*, 1998). These data suggest that in M14 GAS, the transcription of *ahpC, gpoA* and *mrgA* is not induced under conditions of peroxide stress, and is not repressed by PerR (King, Horenstein and Caparon 2000). Primer extension analysis and DNase 1 protection assays indicate that the transcriptional regulator PerR binds to a single promoter upstream of *ahpC* (Brenot, King and Caparon 2005). The regulation of *ahpC* is growth phase dependent and independent of PerR (Brenot, King and Caparon 2005). MrgA is regulated by PerR and plays a key role in oxidative stress resistance in *S. mutans* (Yamamoto *et al.*, 2000), *B. subtilis* and *S. aureus* (Chen and Helmann 1995). In contrast to a previous report (King, Horenstein and Caparon 2000), quantitative real-time PCR analysis of mid-log phase wild-type and *perR* mutant cultures for serotype M14 GAS strain HSC5 revealed higher *mrgA* transcripts for the *perR* mutant, indicating that PerR directly represses *mrgA* transcription through the binding of PerR to the *mrgA* promoter region (Brenot, King and Caparon 2005). PerR is required for full virulence in C57Bl/6J mouse models of subcutaneous and systemic intraperitoneal mouse infections, and a zebrafish model of intramuscular infection (Brenot, King and Caparon 2005).

### PerR regulation of GAS DNases

GAS DNases Sda1, Spd, MF3 and SpnA are important virulence factors (Iwasaki, Igarashi and Yutsudo 1997; Aziz *et al.*, 2004; Buchanan *et al.*, 2006; Chang *et al.*, 2011). Mitogen factor 3 (MF3) and streptodornase 1 (Sda1) are bacteriophage-encoded, whereas Spd and SpnA are chromosomally encoded (Hasegawa *et al.*, 2002; Sumby *et al.*, 2005). PerR directly binds to the promoter region and positively regulates the expression of MF3, a secreted DNase and virulence factor in serotype M1 GAS (Hasegawa *et al.*, 2002; Wen *et al.*, 2011). The expression of *sda1*, encoding for the bacteriophage-encoding DNase Sda1, is up-regulated under oxidative stress conditions in wild-type M1 GAS bacteria, but not in a *perR*-deficient mutant. Gel mobility shift assays revealed that PerR directly binds to the *sda1* promoter region. Mutation of a PerR metal binding site (histidine-99) reduced *sda1* expression in GAS pretreated with H_2_O_2_ (Wang *et al.*, 2013). The PerR-dependent expression of *sda1* may facilitate GAS evasion of the host innate immune response. Sda1 expression enhances GAS degradation of NETs, promoting neutrophil survival and systemic dissemination of GAS from the infection site to normally sterile sites (Walker *et al.*, 2007; Cole *et al.*, 2011). Sda1 also prevents Toll-like receptor 9 recognition of degraded bacterial DNA to promote GAS immune escape (Uchiyama *et al.*, 2012). In M3 GAS, the expression of the phage-encoded DNase MF4 (spyM3_1095) is PerR dependent and induced under conditions of oxidative stress and DNA damage (Banks, Lei and Musser 2003).

### Transcriptional regulator Rgg/RopB

Rgg, also known as RopB, is a DNA-binding global transcriptional regulatory protein that plays a key role in coordinating the expression of cell wall-associated and secreted virulence factors (Chaussee, Ajdic and Ferretti 1999, Chaussee *et al.*, 2001, 2002), secondary amino acid metabolic enzymes (e.g. arginine and serine catabolism) (Chaussee *et al.*, 2003), and proteins involved in thermal and oxidative stress resistance in GAS. The Rgg of GAS is a 280 amino acid (∼33.2 kDa) polypeptide with an N-terminal helix–turn–helix motif that binds to the promoter regions of Rgg-regulated genes (Neely *et al.*, 2003; Anbalagan *et al.*, 2012). In serotype M49 GAS strain NZ131, Rgg regulates the expression of several secreted GAS virulence factors including cysteine protease SpeB, C5a peptidase, M protein, cytolysin streptolysin O and streptokinase (Lyon, Gibson and Caparon 1998; Chaussee, Ajdic and Ferretti 1999; Chaussee *et al.*, 2001, 2002). Inactivation of *rgg* affects the expression of several transcriptional regulatory genes, including *fasBCA, mga, sagA* and *covRS/csrRS* (Chaussee *et al.*, 2002), indicating that Rgg interacts directly or indirectly with other global transcriptional regulators.

Chaussee, Callegari and Chaussee (2004) used a comparative proteomic approach to identify the Rgg-regulated cytoplasmic proteins from mid-log and stationary phase cultures of wild-type serotype M49 GAS and an isogenic *rgg* mutant strain. Cytoplasmic proteins were identified by 2D gel electrophoresis and tandem mass spectrometry and revealed a growth phase-dependent Rgg regulation of proteins associated with arginine metabolism (ArcABC), histidine (HutI) and serine (SdhA) in the exponential growth phase (Chaussee, Callegari and Chaussee 2004). Thermal and oxidative stress response proteins, including ClpE and ClpL, were expressed in the *rgg* mutant, but not wild-type (Chaussee, Callegari and Chaussee 2004). Compared to wild-type, the Rgg-deficient strain was more resistant to heat-shock and puromycin (Chaussee, Callegari and Chaussee 2004), an aminoacyl-tRNA analog that inhibits protein synthesis and induces a heat-shock-like response in Gram-negative and Gram-positive bacteria (VanBogelen and Neidhardt 1990; Frees and Ingmer 1999; Steiner and Malke 2001). The *rgg* mutant was also hypersensitive to killing by superoxide free radicals induced by 50 mM paraquat exposure (Chaussee, Callegari and Chaussee 2004). The oxidoreductases AhpC (SPy2079) and Nox1 (SPy2080) were more abundant in the *rgg*-deficient strain, indicating the de-repression of enzymes associated with GAS resistance to oxidative stress (Chaussee, Callegari and Chaussee 2004).

More recently, a serotype M49 GAS strain deficient in Rgg decomposed more H_2_O_2_ and was more resistant to 4 mM H_2_O_2_-mediated killing, compared to the wild-type parental strain (Pulliainen *et al.*, 2008). A double mutant deficient in *rgg* and *perR* was resistant to 4 mM H_2_O_2_ and did not exhibit changes in the expression of PerR target gene Dpr/MrgA, compared to the *perR* mutant, suggesting that Rgg H_2_O_2_ regulation may be independent of the PerR regulon (Pulliainen *et al.*, 2008). Transcriptomic analysis revealed that the *ahpCF* operon expression was up-regulated in *rgg* mutant GAS (Pulliainen *et al.*, 2008), suggesting that *ahpCF* is regulated by Rgg. The *ahpCF* gene products are involved in reducing organic peroxides, decomposing H_2_O_2_ and enhancing resistance against oxidative stress (Ellis and Poole 1997; Poole *et al.*, 2000). In addition to *ahpC*, serine protease and chaperone HtrA, also known as DegP, is up-regulated in *rgg* mutant GAS (Chaussee, Callegari and Chaussee 2004; Dmitriev *et al.*, 2006). The H_2_O_2_-resistant *rgg* mutant was more virulent in a mouse model of systemic GAS infection (Pulliainen *et al.*, 2008).

### Two-component regulator Ihk-Irr

Voyich and coworkers (Voyich *et al.*, 2003) reported the discovery of Ihk-Irr, a two-component global gene regulatory system that enhances GAS resistance to neutrophil killing and facilitates the lysis of host cells at the site of infection. Upon exposure of GAS to human neutrophils, 276 genes (∼16% of the M1 GAS genome) were differentially transcribed (Voyich *et al.*, 2003). Eleven GAS virulence genes were up-regulated including *sic* (streptococcal inhibitor of complement), *speH* (streptococcal pyrogenic exotoxin H), *ndoS* (endoglycosidase S), *smeZ* (streptococcal mitogenic exotoxin Z) and *speB* (streptococcal pyrogenic exotoxin B), which are known to contribute to neutrophil resistance or modulation of the human innate immune system (Lukomski *et al.*, 1998; Lei *et al.*, 2001; Hoe *et al.*, 2002). GAS genes encoding proteins involved in DNA repair and resistance to ROS-mediated cell damage were up-regulated, including *bsa* (glutathione peroxidase), *ahpC* (alkylhydroperoxidase), *dnaK* (Hsp70) and *nox1* (NADPH oxidase) (Voyich *et al.*, 2003). Phagocytosis of GAS by neutrophils induced the up-regulation of nine genes encoding for proteins that participate in cell wall biogenesis, perhaps in response to the cell wall damage induced by phagocytosis and ROS (Voyich *et al.*, 2003). Metabolic pathway genes were also up-regulated, presumably because an increase in energy metabolism is essential for GAS survival during phagocytosis. Phagocytosis altered the expression of genes encoding for proteins involved in the global transcriptional regulation of GAS. The *mga* regulon, encoding for a transcriptional regulator of virulence factors (Kihlberg *et al.*, 1995), was up-regulated during phagocytosis. The genes encoding the two-component gene regulatory system, *ihk* and *irr*, were up-regulated, suggesting a role for this regulatory system in host innate immunity resistance (Voyich *et al.*, 2003). A serotype M6 GAS strain JRS4 lacking the *irr* gene was hypersensitive to killing by human neutrophils following phagocytosis, indicating that Ihk-Irr plays a key role in the expression of genes necessary for GAS to survive phagocytosis. The *irr* gene is highly expressed in humans with GAS pharyngitis, as assessed by quantitative real-time PCR, suggesting that Ihk-Irr is important for GAS survival in humans (Voyich *et al.*, 2003).

Microarray analysis comparing the global gene expression profiles of a wild-type M6 GAS and a corresponding *irr* mutant demonstrated that Ihk-Irr controls the expression of ∼20% of genes in the GAS genome (Voyich *et al.*, 2004). Several genes involved in oxidative stress resistance were differentially regulated by *ihk-irr*, including *nrdH* (glutaredoxin), *trx* (thioredoxin), *trxR* (thioredoxin reductase), *nox* (NADH peroxidase), *rnr* (ribonucleotide reductase) and *bsaA* (glutathione peroxidase) (Voyich *et al.*, 2004). Multiple virulence-associated genes, including *fbp* (putative fibronectin-binding protein-like protein A), *mf* and *mf3* (DNases), and *sagA* (SLS), and multiple genes involved in cell wall biosynthesis were regulated by the Ihk-Irr regulon (Voyich *et al.*, 2004). The up-regulation of cell wall biosynthesis genes may protect the bacteria from cationic neutrophil antimicrobial peptides such as LL-37 and cathepsin G, which disrupt the bacterial cell membrane (Ganz *et al.*, 1985; Peschel *et al.*, 1999, Poyart *et al.*, 2003). During phagocytosis bacteria are exposed to ROS and antibacterial granule components stored within phagocytic vacuoles. Real-time PCR transcript analysis indicated that *ihk* and *irr* expression are induced upon GAS exposure to H_2_O_2_ and neutrophil primary granules (Voyich *et al.*, 2004). In contrast to wild-type GAS, Irr-deficient GAS were more rapidly killed after phagocytosis through enhanced sensitivity to solubilized neutrophil primary granules, which are composed of α-defensins, elastase and cathepsin G (Sorensen *et al.*, 1997). The *irr*-deficient strain was more susceptible than wild-type to killing by H_2_O_2_ across a range of different concentrations, and was hypersensitive to killing by the cell envelope active antimicrobial peptides LL-37 and cathepsin G. Inactivation of *irr* delayed the formation of skin abscesses and resulted in smaller abscesses, indicating that Irr is required for full GAS virulence in a mouse model of subcutaneous infection. In a bacteremia model of infection, *irr* mutant bacteria were more rapidly cleared from the blood 24 h post-infection compared to wild-type, indicating that Ihk-Irr plays an important role in GAS pathogenesis (Voyich *et al.*, 2004).

### Transcriptional regulator MtsR

Iron is important for many bacterial metabolic functions including the electron transport chain and DNA synthesis repair. However, excess iron is potentially lethal to the bacterial cell through the generation of highly toxic oxygen radicals by the Fenton reaction. In order to maintain iron homeostasis, bacteria express metal-dependent transcription regulators belonging to the Fur or the DtxR family (Andrews, Robinson and Rodriguez-Quinones 2003). GAS acquires iron from heme, hemoglobin, haptoglobin–hemoglobin complexes, ferritin, myoglobin and catalase but not transferrin or lactoferrin (Francis, Booth and Becker 1985; Eichenbaum *et al.*, 1996; Bates *et al.*, 2003). The GAS multimetal transport system (*mts*) is involved in the acquisition of manganese, zinc and iron (Janulczyk, Pallon and Bjorck 1999, 2003; Ge and Sun 2014), and is essential for GAS growth in metal-restricted media and full virulence in a mouse model of GAS infection (Janulczyk, Ricci and Bjorck 2003). Transcriptional regulator MtsR is a member of the DtxR family of metal-dependent regulatory proteins involved in the coordination of iron homeostasis, oxidative stress resistance and virulence (Jakubovics, Smith and Jenkinson 2000). Analysis of an *mtsR* mutant in serotype M49 GAS strain NZ131 by western blot and RNA analysis revealed that constitutive transcription of the 10-gene *sia* (streptococcal iron acquisition) operon (Bates *et al.*, 2005), encoding for the hemoprotein receptor Shr, heme-binding protein Shp (Lei *et al.*, 2002b) and an ABC transporter, mediates GAS iron uptake from hemoproteins (Bates *et al.*, 2003). Electrophoretic mobility gel shift assays indicated that MtsR directly binds to the *sia* promoter in an iron- and manganese-dependent manner to repress the expression of the *sia* operon (Bates *et al.*, 2005). Excess iron accumulation causes oxidative stress via the Fenton reaction (Ratledge and Dover 2000; Touati 2000; Bates *et al.*, 2003). The *mtsR* mutant accumulated more intracellular iron compared to wild-type in ^55^Fe uptake assays in complete medium, and was hypersensitive to H_2_O_2_, indicating that MtsR plays a role in GAS resistance to oxidative stress (Bates *et al.*, 2005). In zebrafish models of intramuscular and intraperitoneal GAS infection, the *mtsR* mutant was attenuated for virulence (Bates *et al.*, 2005).

### Two-component regulator CiaRH

CiaRH is a two-component regulator of GAS gene expression that is up-regulated under conditions of oxidative stress (Riani *et al.*, 2007). Similar to the function of the sensor kinase CiaH in *S. pneumoniae*, the CiaH (Spy_1236) in serotype M1 GAS strain 1529 promotes growth under acidic conditions (pH 6.0) and resistance to oxidative stress following exposure to supraphysiologic concentrations of H_2_O_2_ (61 mM) for 15 min at room temperature (Tatsuno *et al.*, 2014). Pneumococcal CiaH directly up-regulates HtrA, which is involved in resistance to oxidative stress, as described above (Seol *et al.*, 1991; Ibrahim *et al.*, 2004). However, in serotype M1 GAS, the expression of HtrA in the *ciaH*-null mutant was not down-regulated compared to wild-type, suggesting that the contribution of the CiaH sensor kinase to oxidative stress resistance may not be mediated via HtrA (Tatsuno *et al.*, 2014).

## CONCLUDING REMARKS

Bacterial pathogens have evolved a plethora of sophisticated defense mechanisms to counter oxidative damage and highly toxic ROS generated from atmospheric oxygen and the oxidative burst from phagocytes. ROS, including H_2_O_2_, hydroxyl radicals and superoxide anions, are capable of damaging proteins, DNA, membrane lipids, and may induce cell death (Nunoshiba *et al.*, 1999; Storz and Imlay 1999; Imlay 2008). Neutrophils are the first line of defense for the host innate immune system and promote bacterial clearance at the infection site through phagocytosis, a process whereby bacteria are killed by ROS and microbicidal granule components stored within phagocytic vacuoles (Mayadas *et al.*, 2014). Bacterial ROS resistance mechanisms include direct detoxification of harmful reactive oxygen molecules by enzymes (catalases, peroxidases and Sods), repair mechanisms and alteration of intracellular metal ion concentrations (Faulkner and Helmann 2011). GAS resistance to ROS generated by the human innate immune response enables this preeminent human pathogen to survive in the human host under the harsh conditions of oxidative stress.

Compared to other bacterial pathogens, GAS is equipped with surface-associated and secreted factors and unique molecular mechanisms to promote aerotolerance and combat ROS-induced stress *in vitro* and *in vivo*. While GAS lacks catalase, it has evolved additional mechanisms to defend against oxidative stress, including 1) novel surface and secreted molecules (M protein, HA capsule, Mac-1/IdeS and Mac-2); 2) enzymes directly involved in peroxide or superoxide detoxification (SodA, AhpC, GpoA and NoxA); 3) enzymes involved in the repair of ROS-damaged protein or DNA (HtrA/DegP and PolA1); 4) transporters involved in the maintenance of metal ion homeostasis (PmtA, Dpr, MtsABC/SiaABC and Shr); and 5) ROS response regulators (PerR, Rgg/RopB, Ihk-Irr, MtsR and CiaRH).

In Gram-positive bacteria, peroxide-sensing transcriptional regulators are responsible for regulating the oxidative stress response (Imlay 2008; Dubbs and Mongkolsuk 2012). The peroxide-sensing transcriptional regulator, PerR, is the chief peroxide responsive regulator in GAS (King, Horenstein and Caparon 2000; Ricci, Janulczyk and Bjorck 2002; Grifantini *et al.*, 2011). Future work is needed to increase our understanding of the mechanisms of ROS resistance, the complex regulatory networks that coordinate GAS–host interactions and the response to ROS, and how these contribute to GAS pathogenesis and human infection. In addition, many of the proteins involved in ROS resistance are virulence factors. Therefore, these proteins are potential targets for the development of novel anti-GAS therapeutics and immune-boosting agents for the prevention and treatment of streptococcal diseases.
